# Life course learning experiences and infant feeding practices in rural Rwanda

**DOI:** 10.1111/mcn.13126

**Published:** 2021-01-06

**Authors:** Jeanine Ahishakiye, Lenneke Vaandrager, Inge D. Brouwer, Maria Koelen

**Affiliations:** ^1^ Department of Human Nutrition and Dietetics, College of Medicine and Health Sciences University of Rwanda Kigali Rwanda; ^2^ Health and Society Chair Group Wageningen University Wageningen The Netherlands; ^3^ Division of Human Nutrition and Health Wageningen University Wageningen The Netherlands

**Keywords:** breastfeeding, complementary feeding, family influences, infant and child nutrition, infant feeding, qualitative methods

## Abstract

Most studies about infant and young child feeding (IYCF) practices are often perceived as an individual choice depending on mothers' or caregivers' knowledge or attitudes and are focused on mothers' failure rather than successes in adequately feeding their children. However, the role of life course experiences in IYCF is less investigated. Applying a Salutogenic Model of Health, this study on 14 mothers looks at women's life course learning experiences shaping appropriate IYCF practices during the first year of child's life in a rural district of Rwanda. Transcripts from in‐depth interviews were analysed using thematic analysis. Results indicate that positive social interaction with parents or grandmothers during childhood such as sharing meals, parental role models for dietary choices and cooking skills gained by participating in household food preparation played a role in shaping appropriate IYCF practices. Negative experiences during childhood also had a positive influence on IYCF practices for some participants by converting life course constraints into learning opportunities. Motherhood increased mothers' sense of responsibility over their children's health and nutrition. Moreover, mothers' participation in community cooking classes and role modelling approach were strong avenues that enabled their learning through positive interactions and encouragement. Nutrition promotion interventions should consider tailoring nutrition advice to the complexity of mothers' life course experiences by creating opportunities for positive learning experiences of appropriate IYCF practices.

**Key messages**

- Supporting mothers for appropriate IYCF practices requires consideration of their immediate and changing environment.
- Life course experiences including both positive and negative past food upbringing, social interactions with parents or grandmothers during childhood and parental role models for dietary choices play a role in shaping subsequent appropriate IYCF practices.
- The sense of responsibility over child's health and participation in community cooking classes during motherhood are powerful experiences in shaping IYCF practices.
- Findings highlight the need for nutrition interventions engaging mothers in a reflective process on their lived experiences rather than purely scientific food‐health knowledge.

## 1 INTRODUCTION

Optimal infant and young child feeding (IYCF) practices have great potential for reducing child malnutrition and thereby contributing to a reduction of child mortality rate (Black et al., 2008). Many factors interact to determine how a mother feeds her child. IYCF practices are often perceived as an individual choice depending on mothers' or caregivers' knowledge, attitude or belief (van Woerkum & Bouwman, 2014), and most IYCF studies focus on exploring why mothers or caregivers fail rather than on why some mothers or caregivers manage to have healthy IYCF practices (Ramani et al., 2019). IYCF practices (unhealthy and healthy) are learned, supported and expressed within the dynamics of everyday context in which mothers live (Ahishakiye et al., 2019). Past life experiences are known to direct how people make food choices and decisions in the future (Bisogni, Jastran, Seligson, & Thompson, 2012; Devine, 2005). However, the interaction between individuals (in this case mothers) and their environment and the way in which experiences throughout the life course play a role in mother's decision‐making to manage optimal IYCF practices are less investigated.

The Salutogenic Model of Health (SMH) developed by Antonovsky (Lindström & Eriksson, 2010) can support addressing the above knowledge gap. The SMH acknowledges the active role of people in creating health and that health develops from the interaction between people and their everyday life context (Mittelmark, Bull, & Bouwman, 2017). Antonovsky's view of Salutogenesis began with his findings that some people stay healthy despite the influence of many risk factors. He made this observation following a study of female Holocaust survivors, some of whom were found to be well adapted, despite the severe experience in concentration camps and poor life conditions after immigration to Israel (Antonovsky & Sagy, 2017). His question was not why some of these women felt miserable (i.e. what causes disease?) but rather how some of them managed quite well (i.e. what creates health?). Antonovsky viewed health not as a state but as a dynamic move along a continuum, referred to as the ease/dis‐ease continuum (Antonovsky, 1987). Along the life course, human beings are confronted by stressors (challenges) and learn to identify and apply resources that enable them to cope in either a health‐promoting or health‐damaging manner (Lindström & Eriksson, 2010). If people deal successfully with the stressors, they can maintain their health or move towards the ease end of the continuum. Unsuccessful coping with stressors can lead people to a movement towards the dis‐ease end of the continuum (Antonovsky, 1987; Lindström & Eriksson, 2010). The SMH has two central concepts: generalized resistance resources (GRRs) and sense of coherence (SOC) (Idan, Eriksson, & Al‐Yagon, 2017). The GRRs are resources within the individuals (e.g. attitudes, self‐efficacy and knowledge) or in their environment (e.g. social support) that can be used to counter or to cope with the stressors of everyday life. The GRRs provide one with sets of meaningful, coherent life experiences that are characterized by consistency, a balance between overload and underload and participation in shaping the outcome (Vinje, Langeland, & Bull, 2017). The ability to recognize and use these resources is the meaning of the second central concept: the SOC (Eriksson, 2017). Individuals with a strong SOC are better able to identify resources and use them in a health‐promoting way. Those with a strong SOC have the feeling that life is comprehensible, manageable and meaningful. Life experiences are defined as experiences when people learn how to deal with life in general and acquire problem‐solving skills that help to shape one's SOC (Vinje, Langeland, & Bull, 2017). Life experiences characterized by consistency, an underload–overload balance of stimuli and participation in shaping outcomes, contribute to the development of a strong SOC and allow one to reach out in any situation and use the resources to deal with stressors (challenges) (Vinje, Langeland, & Bull, 2017).

In line with the SMH, the central question in this study is, ‘why do some women manage to feed their children well despite their poor living conditions?’ Because of the role of life experiences within the SMH, it is important to consider the life course perspective when studying food‐related behaviours, such as IYCF practices. From a life course perspective, the sequence of socially defined events and roles over time plays an important role (Kok, 2007). The life course research approach takes into account the history and path dependency (the dependency of later developments on previous ones) of the development of practices over time and links the practices to the dynamics of the social and cultural environmental contexts in which the individuals live (Aschemann‐Witzel, 2013). Life course research applied to nutrition‐related health practices helps to understand how past experiences interact with current food practices or choices (Devine, 2005; Devine, Connors, Bisogni, & Sobal, 1998). Hence, the life course of mothers has consequences on their food practices. For instance, a mother's life experiences during childhood and adulthood may have an influence on the way she manages to provide adequate nutrition to her children. Yet little research is available that documents life course experiences driving appropriate IYCF practices among mothers living under adverse circumstances. Such an understanding could be of substantial practical value to health promotion practitioners, in revealing existing contextual influences that promote good child nutrition. Apart from the practical value, such understanding can have a scientific value by providing knowledge about the salutogenic factors or mechanisms indicating the way in which individual mothers deal with their context to manage appropriate IYCF practices. The aim of this study was to gain insights into mothers' life course learning experiences that play a role in shaping appropriate IYCF practices during the first year of child's life in a rural district of Rwanda.

## 2 METHODS

### 2.1 Study site

This study was conducted in the catchment areas of Rutobwe and Buramba health centres located in a rural part of Muhanga District, approximately 49 km south from Rwanda's capital city, Kigali. Muhanga is one of the districts affected by the high levels of stunting among children under 5 years old. The Rwanda Demographic and Health Survey 2014/2015 found that 41.6% of children under age 5 were stunted in Muhanga District, slightly higher than the national average of 38% (National Institute of Statistics of Rwanda, Ministry of Health Rwanda, & ICF International, 2015). The majority of population in Muhanga District live in rural area. The level of poverty remains high with 30.5% of population below the poverty line. The main source of income and food in Muhanga District is agriculture. The main crops are beans, sweet potatoes, cassava, maize, banana, soybeans and potatoes. In terms of gender equity, females spend far more hours on domestic duties than males. Also, females are more occupied in small‐scale farm work than males and less involved in other types of employment. The study was conducted between December 2018 and February 2019.

### 2.2 Participants

Our study is part of the larger longitudinal research that involved 36 mother–child pairs to explore actual breastfeeding and complementary feeding practices and influencing factors from birth until 1 year. Table 1 provides a brief description of the larger longitudinal study.

**TABLE 1:** Socio‐demographic characteristics of women in the larger longitudinal study

Characteristic	Total (*n* = 36)
Age of the mother (years)	
<21	2
21–30	12
>30	22
Marital status (with partner)	32
Ability to read and write	34
Education level of the mother	
Illiterate	2
Primary incomplete	17
Primary complete	15
Secondary incomplete	2
Main occupation (farming)	36
Average number of children	2.3

Mothers' age in the larger study ranged from 18 to 40 years, with a mean age of 34 years. Most mothers (89%) were married. The main occupation for all was farming. Ninety‐four per cent had the ability to read and write their mother tongue. Five per cent had not attended any formal education, 47% did not complete primary school and 42% completed primary school.

In this study, participants consisted of a specific group of 14 mothers ‘doing well’ out of the 36 mother–child pairs. The selection was based on two criteria: (1) following recommended IYCF practices and (2) good growth of the child (growth according to the World Health Organization growth standards) at 1 year of age. The recommended IYCF practices included initiation of breastfeeding within 1 h of birth, exclusive breastfeeding for the first 6 months, continued breastfeeding at 1 year and timely introduction of complementary foods at 6 months. Anthropometric measures that were collected as part of the larger study were used to check that the children were growing well. Children's recumbent lengths were taken at birth and then monthly up to 12 months post‐partum following standardized procedures. Height‐for‐age difference defined as the difference in observed height and median height of a child of the same age and sex from the World Health Organization 2006 growth standard (Leroy, Ruel, Habicht, & Frongillo, 2015) was calculated. Using individual linear regression with height‐for‐age difference as dependent and age as independent variable, a child was qualified as positive grower when having a beta‐coefficient being zero or significantly (*p* < 0.05) positive reflecting growth according or above the standard. A total of 14 mothers complied with the above two criteria, and their respective community health workers confirmed the selected 14 months as ‘doing well’.

### 2.3 Data collection

Narrative inquiry consisting of systematically listening to people's life stories and providing access to peoples' life experiences (Keats, 2009) was used to collect information about participants' life course experiences. Specifically, ‘Food‐Life‐Story’ narrative inquiry that recognizes the active role of people in constructing their own life and their eating practices was used to further map out specific life experiences and how people reflected on these experiences in relation to their food practices (Mittelmark, Bull, & Bouwman, 2017). One visual method, the timeline, was used as a form of graphic elicitation to encourage the construction of rich temporal stories on how one's pasts shape presents (Sheridan, Chamberlain, & Dupuis, 2011). Data were collected through two consecutive sequences of interviews: preliminary and in‐depth interviews. During the preliminary interviews, participants were given details on how to draw their timeline representing their key life events and past experiences. Participants were asked to think back and to create their personal chronological timeline in which they were free to include all relevant aspects of their life trajectories, key moments and past life experiences with food and child feeding practices. The same opportunity was used to check participants' demographic and socio‐economic characteristics. During the follow‐up in‐depth interviews centred on their timelines, participants were asked to talk about their life experiences. By use of probing questions, the timeline served as a tool for respondents to tell about their life stories. For instance, why is this life experience important to you? Can you describe what happened? What has this life experience brought to you referring to your child feeding? How does this life experience help you or not to comply with the recommended practices? How did you learn this compliance?

### 2.4 Data analysis

The interview recordings were transcribed verbatim and translated from Kinyarwanda into English. Each participant was assigned an identity to guarantee anonymity. Atlas.ti software was used for coding and analysing the data. Analysis across all transcripts was done thematically following the protocol described by Braun and Clarke (2006). The first step of analysis consisted of reading and re‐reading of the transcripts to familiarize with the data. Second, researchers generated initial codes within the transcripts in the left side margin of the transcripts. The initial coding was done by the first author. The second author also coded independently half of the transcripts. Codes were compared and discussed by the first and the second authors to ensure that the data segments were categorized correctly. The two researchers' categorization was almost identical. Any inconsistencies were discussed with the fourth author until agreement was reached. The coding was guided by the research question on life experiences that play a role in shaping appropriate IYCF practices. Potential themes were formed from initial codes and reviewed for their relevance in relation to the coded extracts and entire dataset. Examples of these themes are key learning moments and life experiences through the life course highlighted by participants as significantly influencing appropriate feeding practices. The findings were further discussed and challenged in a series of team meetings involving all authors, from which further analytical refinements emerged.

### 2.5 Ethical considerations

The study was approved by the institutional review board of the College of Medicine and Health Sciences in Rwanda (approval notice: No. 058/CMHS IRB/2016). Written informed consent was obtained from all participants.

## 3 RESULTS

**FIGURE 1:**
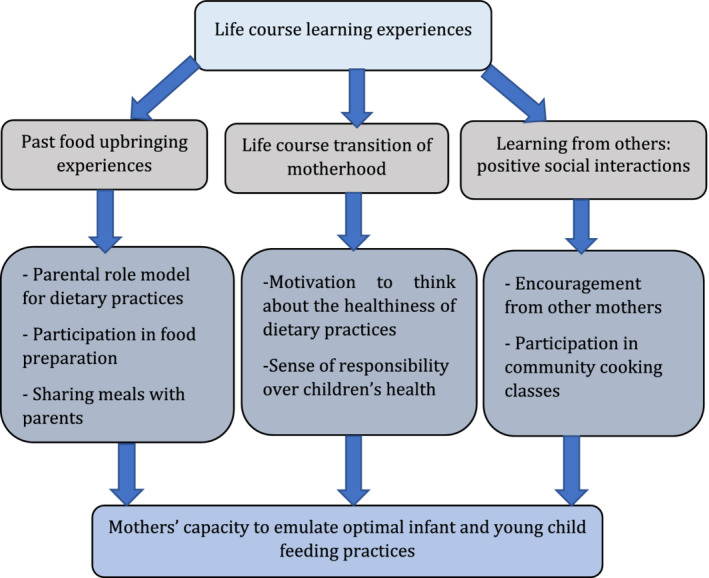
Summary of the main results

This section starts with a brief description of the participants' characteristics, followed by key moments or events on the timelines as indicated by participants. Additionally, as represented in Figure 1, the section elaborates on past food upbringing experiences, the influence of motherhood as a major life course transition and the learning from others through positive interactions.

### 3.1 Characteristic of the study participants

Table 2 presents the participants' characteristics. Their age ranged from 24 to 40 years, with a mean age of 34 years. Most participants were married. All participants, except one, had the ability to read and write. Less than a half of the participants completed primary school education (six out of 14). The main occupation was agriculture for all participants. The majority of participants had three or more children.

**TABLE 2:** Socio‐demographic characteristics of the study participants

S/N	Participant code	Age of the mother	Marital status	Ability to read and write	Education level	Main occupation	Number of children
1	KI	33	With partner	Yes	Primary complete	Agriculture	3
2	MB	25	With partner	Yes	Primary incomplete	Agriculture	1
3	MJ	33	With partner	Yes	Primary incomplete	Agriculture	3
4	MV	36	With partner	Yes	Primary complete	Agriculture	3
5	MMT	38	With partner	Yes	Primary complete	Agriculture	3
6	MC	28	With partner	Yes	Primary complete	Agriculture	2
7	NA	32	With partner	Yes	Primary incomplete	Agriculture	2
8	NMC	25	No partner	Yes	Primary complete	Agriculture	1
9	TJ	31	With partner	Yes	Primary incomplete	Agriculture	3
10	UG	39	With partner	Yes	Primary incomplete	Agriculture	4
11	UE	26	With partner	Yes	Secondary incomplete	Agriculture	3
12	UY	41	With partner	Yes	Primary incomplete	Agriculture	3
13	YC	41	With partner	No	Illiterate	Agriculture	6
14	YE	38	With partner	Yes	Primary complete	Agriculture	4

### 3.2 Key moments or events on the timelines as indicated by participants

All participants indicated key moments (events) and experiences during their life course that influenced their food practices. Most timelines started from the participants' early childhood until motherhood. The narrated moments or events varied from one participant to another. Important moments or events reported as influencing food practices included

- death of mother, father or both parents;
- living with grandmother or stepmother;
- responsibility as family cook at young age;
- taking care of older sisters' or neighbours' babies at young age;
- sharing meals with siblings or parents;
- parental examples of preparing foods, both positive (e.g. variety of foods, including vegetables) and negative (monotonous meals); and
- life course transitions such as getting married, pregnancy and childbirth.

Both positive and negative life experiences and events played a role in shaping the participants' IYCF practices. For instance, childhood events such as the death of the father or the mother and being raised by stepmother gave rise to stressful situations because they used to eat monotonous diets and sometimes with food scarcity during childhood. However, later in their life course, some mothers managed to convert those life course constraints into learning opportunities that motivated them to work very hard to be able to feed their children differently.

### 3.3 Past food upbringing experiences

The influence of dietary practices learnt early in the life course was one major recurring subject throughout most of the interviews. Most participants reported remembering their upbringing with respect to food and emulating some of their parents' food habits as well as what they learned from their upbringing in terms of foods. These dietary practices arose while the participants were living with parents during childhood. The majority of participants mentioned that parental role models for dietary choices and repeated exposure to a varied diet during childhood played a role in their current IYCF practices. For instance, participants who had favourable early experiences with vegetables consumption during childhood described positive trajectories of constant consumption and that they are now emulating the same practices in their current food preparation for infants.

When I was a child and as I grew up, my mother was the one responsible for food preparation. The meals she cooked were always a mixture of different foodstuffs, and vegetables were the principal component. We always ate together, sometimes we lost appetite because of vegetables and she would persuade us to eat telling us vegetables make children grow well and now I imitate my mother's practices and feed my child most of the time a diet containing vegetables. (UE)

Participants reported to learn not only from their mothers but also from their family members including older sisters. For instance, participants reported recalling and emulating their older sisters' breastfeeding practices.

I took care of my elder sister's young child and learnt many things from her when she gave me instructions on what to do for the child. I used to prepare porridge and feed the baby. I valued the instructions about what I used to do for that child, and I did them for my child. (MB)

Other participants reported how their early participation in food preparation played an important role in their current IYCF practices.

I watched how my mother used to prepare meals when I was young, and I started helping her to prepare foods as I grew. Today I imitate what my parent had been doing. (NC)

The participants grew up in different family situations, and therefore, their childhood experiences were varied. Some of the participating mothers reported not to continue the food practices they learned during childhood because they considered their childhood food upbringing as a sort of negative example that triggered them to do differently.

Therefore, I can't tell you that I had a good experience during my childhood. We never ate to our satisfaction, and sometimes we would steal food where our stepmother had hiden it. We experienced such tough conditions, but God protected us. Thus, I did not experience something positive that I can imitate in raising my child. This made me decide to do my best that my children will not experience a shortage of food like what happened to me. (UG)

### 3.4 Major life course transition: Motherhood

Participants discussed how they became more aware of the importance of healthy food and made some changes in their food practices during the transition to motherhood. Motherhood motivated them to think more critically about healthiness of their dietary practices and to make some changes in their dietary practices for the health benefits of their children via one's own diet during pregnancy and the first 6 months of exclusive breastfeeding. In addition, changes in dietary practices happened later when the child started complementary food in order to provide healthy meals to their children and foods adapted to the child's stomach capacity.

For some participants, the unhealthy experiences such as seeing infants in the neighbourhood who have not exclusively been breastfed for 6 months prompted them to adopt exclusive breastfeeding.

I often see infants who have been fed prematurely suffer from intestinal worms and their belly swell up. …. It makes a child sickly. Therefore, I decided to be careful with my children's nutrition. (UG)

For the majority of the participants, motherhood triggered a sense of responsibility for their children's health, taking an active role. This sense of responsibility encouraged mothers to implement the teachings from the health professionals and community health workers, and it increased mothers' obligation and motivation to actively take care of their children including breastfeeding the baby and on time, preparing food by themselves and feeding their children a balanced diet during the complementary food period. Participants felt responsible for their children's health and understood that child feeding plays an important role.

I have to devote a part of my time and spare it for my child. I should have devotion to my child. For instance, during the village kitchen activities, they advise us to use cheap foodstuffs like vegetables and small fishes in our dishes because we cannot afford meat. I try my best to implement the teachings. (UY)

### 3.5 Learning from others: Positive interactions

Support from social networks (female family members, peers and older mothers from the community and mother groups) played a major role for the practice of exclusive breastfeeding for the first 6 months and complementary feeding practices. Many participants reported encouragement from other mothers about exclusive breastfeeding and complementary feeding.

I managed to breastfeed him for 6 months while I had thought that if I gave him food before this age he would grow well. I learnt exclusive breastfeeding from older mothers who practiced breastfeeding. They told me that giving food to an infant before 6 months of age is harmful to its health. (MB)

Most of the participants participated in community cooking classes:

Community health workers sometimes teach us how to prepare food for our children during the village kitchen cooking demonstrations sessions. This programme was launched in all villages. We provide foodstuffs and we prepare a mixture of them and feed our children. I learn some new cooking skills when I am involved in. (MMT)

## 4 DISCUSSION

This study extends the qualitative literature on IYCF by exploring the life course experiences that play a role in shaping appropriate IYCF practices during the first year of a child's life among mothers in a rural district of Rwanda. Also, this study is among the first studies applying the salutogenic approach to explore the role of life course learning experiences on IYCF practices in the context of low‐ and middle‐income countries.

Three major insights emerge from our study, related to (1) past food upbringing experiences, (2) learning from others and (3) transition to motherhood (see Figure 1). Below, we discuss these findings within the framework of the SMH, more specifically in relation to the three dimensions of the SOC: comprehensibility, manageability and meaningfulness.

*Comprehensibility* is the cognitive dimension of SOC. Life experiences that evoked comprehensibility—the extent to which, during the course of growing‐up, messages were clear and there was order and structure rather than chaos in one's environment—were observation of good parental cooking and eating patterns consisting of a variety of foods and participation in household food preparation during childhood. For most of the participants, childhood food upbringing played a role in shaping their subsequent IYCF practices, especially experiences characterized by positive social interaction with their own mothers and family members. Most participants remembered about their food upbringing with respect to basic knowledge and cooking skills they acquired. These findings are in line with a previous study about life course influences on fruits and vegetables trajectories that found that early experiences provide lasting food roots that provide reference points for later comparison (Devine, Connors, Bisogni, & Sobal, 1998). For some participants, unpleasant food experiences during childhood triggered them to do things differently during motherhood for the benefits of their children; that is, they managed to convert life course constraints into learning opportunities. The SMH postulates that people are exposed to events that may be considered as stressors and affect health as they can reduce health temporarily but can also in the long term strengthen people in a way that makes it possible to deal with stress in another situations (Eriksson, 2017). In a study to unravel the mechanisms underlying healthful eating, Swan et al. (2018), concluded that it is never too late to promote healthful eating, because even in the face of adverse experiences, challenges could be overcome later in life.

*Manageability* is the behavioural dimension of SOC. Life experiences that evoked manageability—a belief that the resources are available to manage appropriate IYCF within mothers' own control—included role models of appropriate IYCF and participation in community cooking classes. Participants' involvement in community actions such as participation in community cooking classes was reported to contribute to successful IYCF practices. For many participants, participation in community cooking classes was in the backdrop of their narratives. This was particularly important for an increased capacity in cooking by gaining new food preparation skills as well as an increased awareness of food ingredients readily available in their community for complementary feeding. Role models of appropriate IYCF practices served as a resource for participants in learning and managing appropriate IYCF practices. Previous research has shown that social support had influence on individuals' past and current ways of managing dietary practices (Bisogni, Jastran, Shen, & Devine, 2005). This finding implies that dietary behaviours, for instance, IYCF decisions and actions, are learnt, supported and embedded in the social environment (Ahishakiye et al., 2019).

*Meaningfulness* is the motivational dimension of SOC. Life experiences that evoked meaningfulness—a belief that there is a good reason to maintain or care about their IYCF practices—included participants' sense of responsibility over their children's health and awareness of the benefits of optimal IYCF practices during motherhood. Becoming a mother (motherhood) triggered an increased sense of responsibility over children's health and nutrition and increased their awareness of the benefits of optimal IYCF practices. This made mothers to be more internally motivated. With this internal motivation, healthy food practices, including IYCF practices, become more meaningful to mothers because they are important for the good health of their children. Previous studies found that the health of the child is the most important motive for mothers to manage healthy food practices (Edvardsson et al., 2011; Hingle et al., 2012). Or, as Aschemann‐Witzel (2013) argues, the period around pregnancy and the transition to motherhood have a positive effect on the feeling of responsibility of the child.

The approach in our study, using the life course perspective and applying the SMH, certainly has added value. Whereas several studies examine health‐related behaviours from a life course perspective, few studies have focused on food‐ and nutrition‐related behaviours (Szwajcer, Hiddink, Maas, Koelen, & van Woerkum, 2012). Among them, some have emphasized on specific foods items whereas others have examined food practices of individuals in certain life stages without considering the whole life course of individuals (Aschemann‐Witzel, 2013; Devine, Connors, Bisogni, & Sobal, 1998). Little attention has been paid to examine how health‐related behaviours such as IYCF practices develop over a person's life course and how past experiences interact with current context to shape appropriate IYCF patterns as we did in this study. As premised by Antonovsky (1987), the foundation of individuals' SOC is laid during childhood when children have life experiences that are characterized by an underload–overload balance, consistency and socially valued decision‐making. The use of SMH in this study allowed for further confirmation of this statement. Past food upbringing experiences, learning from others and transition to motherhood contributed to the feeling of mothers that IYCF practices are comprehensible, manageable and meaningful. Parents (family) play an important role in the learning process about appropriate practices of their children, especially positive food upbringing experiences. Also, learning from others (role models) contributes to this. This is in line with a study by Bull, Mittelmark, and Kanyeka (2013), which found that participation in community groups of various kinds build individual women's skills and play a key role in making any activity meaningful, comprehensible and manageable. This in its turn supports mothers to develop their sense of agency and thus gain greater control of their IYCF practices. Social support, from both family and community members, provides an individual with coherent life experiences (Antonovsky, 1979). Finally, sense of responsibility, triggered by motherhood, is a powerful experience. As Szwajcer, Hiddink, Koelen, and van Woerkum (2007) argue, those periods are occasions when women become more aware of the health aspects of nutrition and are more motivated to eat healthy. It is the meaningfulness of the life event or experience (IYCF practices in this case) that determine the person's comprehension as well as their willingness to invest resources to succeed (Thomson & Dykes, 2011). Once individuals are motivated, goal‐directed activities are initiated and maintained (Cook & Artino, 2016), and through those activities, they also learn to identify and use certain resources (GRRs).

### 4.1 Strengths and limitations

One of the strengths of this study was the use of a visual research tool of timelining. The use of timelining elicited self‐reflection and story‐telling among study participants (Sheridan, Chamberlain, & Dupuis, 2011). In such conditions, the interviews were led by what participants marked as important aspects of their past life experiences without interference from the researchers.

This study has some limitations as well. First, the study was limited to a specific group of mothers, from one rural geographical location in Muhanga District, with a minimal sample size (*n* = 14). Therefore, the generalizability of the findings to a different or wider population is low. However, generalizability of the findings was not of primary importance as this study rather aimed to obtain richer and in‐depth accounts in the case studied, which would not have been achievable with a large sample. Second, only mothers who followed the recommended IYCF practices and whose children were growing well were included in the study. Future research could explore similar investigations by considering all mothers of the larger study and classify them into categories based on the criteria of IYCF and child growth (meeting IYCF criteria, meeting growth criteria, meeting IYCF and growth criteria and meeting no criteria) to analyse the different experiences and try to see where patterns emerge. Third, besides nutrition‐related factors like feeding practices, other factors such as genetic factors are related to child growth in height (Addo et al., 2013). Future research should consider mother's height while determining children who are growing well. Also, child's characteristics such as birth order are known to influence breastfeeding and complementary feeding practices (Dhami, Ogbo, Osuagwu, & Agho, 2019; Shiferaw, Mossa, & Gashaw, 2017). However, there are conflicting findings with regard to the consistency of the associations from one setting to another. In our study, the sample size was too small to explore its influence on mothers' feeding practices. Fourth, the questions were mainly focused on nutrition rather than on life in general. Consequently, how women cope in general and how this relates to their IYCF practices were not explored. Future research should explore these questions to gain insights into more general health‐promoting factors along the life course that generates healthy IYCF practices.

### 4.2 Implications

The findings of this study have a number of practical implications for nutrition promotion professionals and programmes:

- Tailoring food and nutrition advice to the complexity of mothers' life course experiences by creating opportunities for mothers to reflect on their lived experiences and the role of past life experiences on how they deal with their daily lives to manage well IYCF practices (mothers‐driven learning) instead of the one‐size‐fits‐all educational approach is fundamentally important.
- Health professionals should also focus on the social environment in which mothers live, because the interaction of the mothers with their social environment is important for appropriate IYCF practices. Facilitating food‐ and nutrition‐directed learning through community actions, including community cooking classes where mothers can practice appropriate practices, is an example.
- Finally, it may be important for health professionals to support mothers in discovering the meaningfulness of IYCF practices.

## CONFLICTS OF INTEREST

The authors declare that they have no conflicts of interest.

## CONTRIBUTIONS

JA designed the study protocol, conducted the in‐depth interviews, coded and analysed the data and wrote the manuscript. LV contributed to the design of the study protocol, analysed the data and guided the analysis. LV, IDB and MK guided the writing of the manuscript, reviewed the manuscript and approved it for submission. All authors read and approved the final manuscript.
